# Note on the Automatic Reduction of Hip-Joint Luxation

**Published:** 1877-08-20

**Authors:** H. H. A. Beach


### NOTE ON THE AUTOMATIC REDUCTION OF HIP-JOINT LUXATION.

BY H. H. A. BEACH, M.D.

In the issue of the *Times* of June 23, 1877, is a letter from New York, dated June 19, 1877, which states that "Dr. A. B. Crosby has just given, at the Academy of Medicine, an exposition of what he terms *the automatic method of reducing luxations of the hip.*" From this letter I extract the following:

After flexion of both thighs, "Dr. Crosby then placed his hands under the calves of the legs, quite near the knees. . . . Raising the pelvis a short distance from the floor, the head of the bone at once slipped in. He explained that in this procedure the patient was made to perform the reduction himself, a sort of *felo-de-se*, as he termed it, the weight of the body supplying the extension, while the counter-extension was made by the operator.

"The method was first described to him by a friend of his in Vermont, Dr. J. G. Allen, who had hit upon it accidentally, about two years ago, while in the act of lifting a patient suffering from this dislocation." . . . "In Dr. Bigelow's admirable monograph on luxation of the hip (a copy of which, strange to say, he found it difficult to lay his hands on in New York), he has found that the same position was used in a number of intances there recorded, but the method pursued was always different from that which he (Dr. Crosby) had ventured to call the automatic."—*Phil. Med. Times*, June 23, p. 447, 448.

In connection with these remarks of Dr. Crosby, I have only to say that it is a little difficult to see why Dr. Crosby flexes and lifts both thighs. He thus loses half of the counter-extending weight of the body.

Dr. Bigelow flexes only the dislocated thigh, and suspends the body by that alone. Otherwise, Dr. Crosby's alleged novelty is identical with the method fully described by D. Bigelow in his monograph. It is the method preferred by him, and I have repeatedly seen it efficient in his hands. It has been also used in England and credited to Dr. Bigelow.

His description of this method is as follows (see "The Hip," H. C. Lea, Philadelphia, 1869, p. 46). As the work is out of print, I have copied it:

"REDUCTION OF THE DISLOCATION OF THE DORSUM.—The dislocation may be equally well reduced by traction or rotation.

"*By traction.* 1. Lay the patient, when etherized, on his back upon the floor, bend the limb at the knee, flex the thigh upon the abdomen, adduct and rotate it a little inward to disengage the head of the bone from behind the socket. The Y ligament is then relaxed. . . . The thigh need only be forcibly lifted or jerked towards the ceiling with a little simultaneous circumduction and rotation outwards, to direct the head of the bone towards the socket.

"2. The surgeon's foot (divested, it need hardly be said, of boot or shoe) may be placed on the anterior superior spinous process of the ilium, or on the pubes, to keep the pelvis down, while he pulls the flexed knee up. Or, in the same way, while assistants suspend the pelvis a few inches from the floor, by a strip of board passed traversely under the calf near the ham, the surgeon may with his foot thrust the pelvis down to its place.

The mere weight of the body is not always sufficient to accomplish the object. Dr. Bigelow elsewhere shows that forcible traction is sometimes required. *Phil. Med. Times.*

CREAM FOR CHILDREN.—Sweet cream diluted in different proportions with boiling water, according to the age of the child, to which add ten or fifteen grains sugar of milk, constitutes an excellent diet in diarrhœas of children

